# Femtosecond laser-assisted *in situ* keratomileusis for the correction of residual ametropia after penetrating keratoplasty: 1-year follow-up

**DOI:** 10.3389/fopht.2025.1562555

**Published:** 2025-04-11

**Authors:** Dario Pasquale Mucciolo, Giancarlo Albani, Luca Terracciano, Marco Branchetti, Laura Luchetti, Vittoria Murro, Gianni Virgili, Fabrizio Giansanti

**Affiliations:** ^1^ Department of Neuroscience, Psychology, Drug Research and Child Health, University of Florence, Florence, Italy; ^2^ SOC Oculistica Prato, Azienda USL Toscana Centro, Firenze, Italy; ^3^ Fondazione GB Bietti, Roma, Italy

**Keywords:** femtolaser LASIK, penetrating keratoplasty, PKP, post-operative ametropia, refractive surgery

## Abstract

**Background:**

After an optimal corneal transplantation, a residual refractive error is possible due to several factors. We evaluated the 1-yr follow up of laser-assisted *in situ* keratomileusis using femtosecond laser (LASIK) for the correction of residual ametropia after penetrating keratoplasty (PK).

**Methods:**

Ten eyes of 10 patients were treated using corneal Femto-LASIK (F-LASIK) (WaveLight® Refractive Suite, Alcon) to correct refractive errors after PK at Careggi Teaching Hospital (Florence, Italy). The main outcomes included uncorrected and corrected distance visual acuity (UDVA, CDVA), preoperative and postoperative manifest refraction, and corneal topography. All patients were evaluated the day after surgery and 1, 4, 12, 24, 48 weeks later.

**Results:**

At the 48-week follow up, all patients showed a significant improvement in their UDVA (mean: 0.95 ± 0.29 LogMAR vs 0.50 ± 0.22 LogMAR, p<0.05) as well as in the spherical equivalent value (SE) (mean: -4.50 ± 2.37 vs. -1.55 ± 0.77, p<0.05), the cylindrical ametropia (mean: -6.13 ± 2.04 vs. -3.20 ± 2.15, p<0.05) and the CDVA also improved (median 0.26 [0.1-0.9] vs 0.22 [0.1-0.4] LogMAR, p<00.05). These values were observed from the 12-week follow up onwards. Post-operative spherical ametropia was not statistically significant. Intraoperative and postoperative complications were not detected.

**Conclusions:**

UDVA significantly improved using Femto-LASIK without surgical complications. The refractive results were stable from the 3-mth to the 1-yr follow ups. Femto-LASIK is an effective and safe choice to treat post-PK refractive errors.

## Introduction

Penetrating keratoplasty (PK) is an effective and safe surgical technique to treat several ocular diseases, although post-surgery visual rehabilitation represents a great challenge for the surgeon. Significant degrees of ametropia (regular and irregular astigmatism, often associated with myopia, less commonly with hyperopia) and/or anisometropia can lead to unsatisfactory refractive outcomes for patients, even if uncomplicated surgery is performed (1–4). After an optimal corneal transplantation, a residual refractive error is possible due to several factors: pre-operative corneal irregularity (involving both the host and the donor tissue), intra-operative tissue alignment, suture technique, suture adjustment or time of suture removal and post-operative wound healing course (5, 6).

The mildest cases can be managed using spectacles or contact lenses; however, in many patients the induced ametropia and/or anisometropia cannot be fully corrected in this way and they require surgical correction (5, 6) especially when an high astigmatism is present (wedge resections, and different incisional keratotomies) (5, 7–14).

Since the 1990s, Photorefractive keratectomy (PRK) has been used after PK, but several studies have shown significative risk of regression and stromal haze that limit its effectiveness (15, 16). Recently, LASIK with Femtosecond laser flap creation has improved the postoperative management of PK patients (16–25); for this reason some authors (26, 27) have suggested using femtosecond laser for flap creation, in order to perform the least invasive treatment, but they reported a small number of cases and short follow ups. The advent of topography-guided ablation systems has made it possible to perform customized treatments and correct irregular astigmatism. However, in many cases, insufficient stromal bed thickness does not allow for full correction of refractive errors (25, 28). In our study we have described the refractive and visual results of 1-stage Femto-LASIK after penetrating keratoplasty performed in a single center using WaveLight Laser suite, Alcon.

## Methods

We retrospectively evaluated the consecutive medical charts of the patients who underwent F-LASIK between January 2017 and June 2018 for residual refractive errors after penetrating keratoplasty. Spectacle or contact lens corrections were not tolerated by the patients (after achieving anatomical and refractive stability).

All surgical procedures (both PK and F-LASIK) were performed at Careggi Teaching Hospital by the same experienced surgeon (A.G.). A comprehensive explanation of the procedure, including the risks associated with F-LASIK technique, were administered to the patients before surgery. The inclusion criteria were as follows: endothelial cell count > 1500 cell/mm^2^, clear full-thickness corneal graft, clear lens (if patient was phakic). Exclusion criteria included the presence of other ocular diseases than the indication for PK, vascularized cornea, simulated keratometry readings below 37 or above 55 diopters, anterior posterior misalignment of the graft–host junction.

Refractive surgery after PK was performed 3 months after total suture removal to be sure of complete wound healing, ideal graft condition and stable refraction. Refraction was checked in two consecutive pre-operative visits, at least 3 weeks apart. The study was approved by the local Ethics Committee (Careggi Teaching Hospital) and adhered to the Declaration of Helsinki; an informed consent was obtained from all participants.

### Pre-operative evaluation

Patients underwent a complete ophthalmological examination including manifest refraction by auto-refraction (KR/RM-800, Topcon), UDVA, CDVA, corneal topography, tomography (Sirius, CSO, Florence, Italy), OCT examination and pachymetry (MS-39, AS-OCT, CSO, Florence, Italy).

UDVA and CDVA were tested under standard lighting conditions using ETDRS visual acuity chart.

### Surgery

WaveLight® Refractive Suite (WaveLight® EX500 Excimer laser + WaveLight® FS200 Femtosecond Laser, Alcon, Fort Worth, TX, USA) was used to create a 110 μm-lamellar flap with a superior hinge. The flap was planned to be a little smaller than the previous corneal graft (flap diameter average: 0.2 mm or less) and was put in the center of the donor button, avoiding the graft-host junction. The flap was then lifted, and excimer laser ablation was carried out. The flap position was checked at the slit-lamp after surgery. A residual stromal bed of 280 μm or more was left in all eyes. After flap repositioning, the postoperative treatment consisted of topical antibiotic-steroid association for 15 days.

### Post-operative evaluation

All patients were evaluated the day after surgery and 1, 4, 12, 24, 48 weeks later. Manifest sphere, cylinder, axis, and manifest spherical equivalent, UDVA and CDVA were evaluated at 3,6 and 12 months. Corneal topography and anterior-segment OCT were performed at 1 month, 6 months, and 1 year.

### Statistical analysis

A paired t-test was used to compare preoperative and postoperative continuous variables. Normality of the data distribution was assessed using the Shapiro-Wilk test. If normality was not met, the Wilcoxon signed-rank test was applied instead. A p-value < 0.05 was considered statistically significant. Results are presented as mean ± standard deviation (SD) or median and min-max range, as appropriate.

## Results

We included in our study 10 eyes of 10 patients who underwent PK between 2014 and 2015 (mean age 58.75; Range 39 - 79). Seven patients were male. All patients except 2 were pseudophakic. The underlying condition leading to PK was keratoconus in all cases examined in our series. Corneal graft sizes ranged from 7.0 to 8.5 mm (mean: 8 mm).

Only 3 patients (3/10) were characterized by cylindrical errors > 6 diopters (-6.13 ± 2.04; range -4.25, -10) (**Table 1**).

**Table 1:** Visual and refractive parameters preoperatively and postoperatively at the last follow-up visit.

ID	Age	UDVA Pre LogMAR	CDVA Pre LogMAR	K1/K2	Pachy pre	Sph eq pre	Sph pre	Cyl pre	UDVA post LogMAR	CDVA post LogMAR	K1/K2 post	Pachy post	Sph eq post	Sph post	Cyl post
1	40	0.70	0.10	43.34/50.26	612	-4.25	-1.25	-6	0.22	0.10	42.85/46.58	593	-0.25	0	-0.5
2	24	0.49	0.22	44.61/48.74	634	-2.75	-0.75	-4.25	0.40	0.10	44.15/45.58	464	-1.75	-0.75	-2
3	76	1.30	0.92	39.32/52.80	573	-9	-6	-6	1.00	0.40	37.99/47.81	484	-3.25	0.75	-8
4	79	1.30	0.40	40.12/49.24	555	-1.75	2.25	-8	0.70	0.40	41.96/45.82	462	-1.5	1	-5
5	72	1.00	0.10	41.81/49.36	593	-4.5	-0.25	-8.5	0.49	0.10	39.53/44.02	543	-1.25	1.25	-4.5
6	70	1.00	0.30	44.87/49.13	513	-8	-6	-4	0.49	0.22	43.21/47.19	384	-2	-0.5	-3
7	69	1.30	0.22	42.53/53.84	498	-5.5	-0.5	-10	0.40	0.22	41.79/47.15	425	-1.5	0	-3
8	48	0.70	0.22	42.87/47.98	547	-3.25	-1	-4.5	0.30	0.16	42.99/44.76	444	-1.75	-1	-1.5
9	39	1.00	0.30	41.13/45.22	581	-3.25	-1	-4.75	0.49	0.22	40.97/43.05	485	-1.25	-0.25	-2
10	70	0.70	0.49	43.25/49.11	569	-2.75	-0.25	-5.25	0.49	0.40	43.01/45.88	453	-1	0.25	-2.5

UDVA, uncorrected distance visual acuity; CDVA, corrected distance visual acuity; Pachy, pachymetry; Sph Eq, spherical equivalent; Cyl, Cylinder.

At the 12-month post-operative evaluation, all patients showed a significant improvement in their UDVA (mean: 0.95 ± 0.29 LogMAR vs 0.50 ± 0.22 LogMAR, p=0.0001; Student’s t-test), as well as in the SE (spherical equivalent) (mean: -4.50 ± 2.37 vs. -1.55 ± 0.77, p=0.001; Student’s t-test), cylindrical ametropia (mean: -6.13 ± 2.04 vs. -3.20 ± 2.15, p=0.004, Student’s t-test) and CDVA (median [min-max], 0.26 [0.1-0.92] vs 0.22 [0.1-0.4], p=0.0273, Wilcoxon signed-rank test). The CDVA showed a statistically significant improvement, but the level of significance is considered weak. These values were stable in the intermediate follow ups, even 3 months after refractive surgery.

Post-operative spherical ametropia was not statistically significant (median [min-max] -0.88 [-6.0 + 2.25] vs 0.0 [-1.0 + 1.25], P= 0.0581, Wilcoxon signed-rank test).

The attempted astigmatic correction was not reached completely in all cases. Mean preoperative and postoperative visual and refractive parameters at the last follow up can be found in **Table 2**. There were no intraoperative or postoperative complications such as corneal wrinkles, epithelial ingrowth, or graft rejection.

**Table 2:** Preoperative vs postoperative parameter comparison.

	PRE	POST	p value
UDVA (mean ± SD)	0.95 ± 0.29	0.50 ± 0.22	0.000127*
CDVA (median [min-max])	0.26 [0.1-0.92]	0.22 [0.1-0.4]	0.0273**
Sph Eq (mean ± SD)	-4.50 ± 2.37	-1.55 ± 0.77	0.00107*
Sph (median [min-max]	-0.88 [-6.0 + 2.25]	0.0 [-1.0 + 1.25]	0.0581**
Cyl (mean ± SD)	-6.13 ± 2.04	-3.20 ± 2.15	0.00412*

UDVA, uncorrected distance visual acuity; CDVA, corrected distance visual acuity; Sph Eq, spherical equivalent; Cyl, Cylinder; * t-student; ** Wilcoxon.

## Discussion

PK is performed to restore full-thickness corneal integrity and vision in several corneal pathologies. However, functional visual quality could be limited because of the resulting high astigmatism, ametropia, or both (29). LASIK has been widely used to treat a huge number of refractive disorders; in fact, after laser ablation in the corneal stroma the postoperative wound healing reaction is very mild, and the risk of haze and scarring development is very low (30, 31).

**Figure 1:**
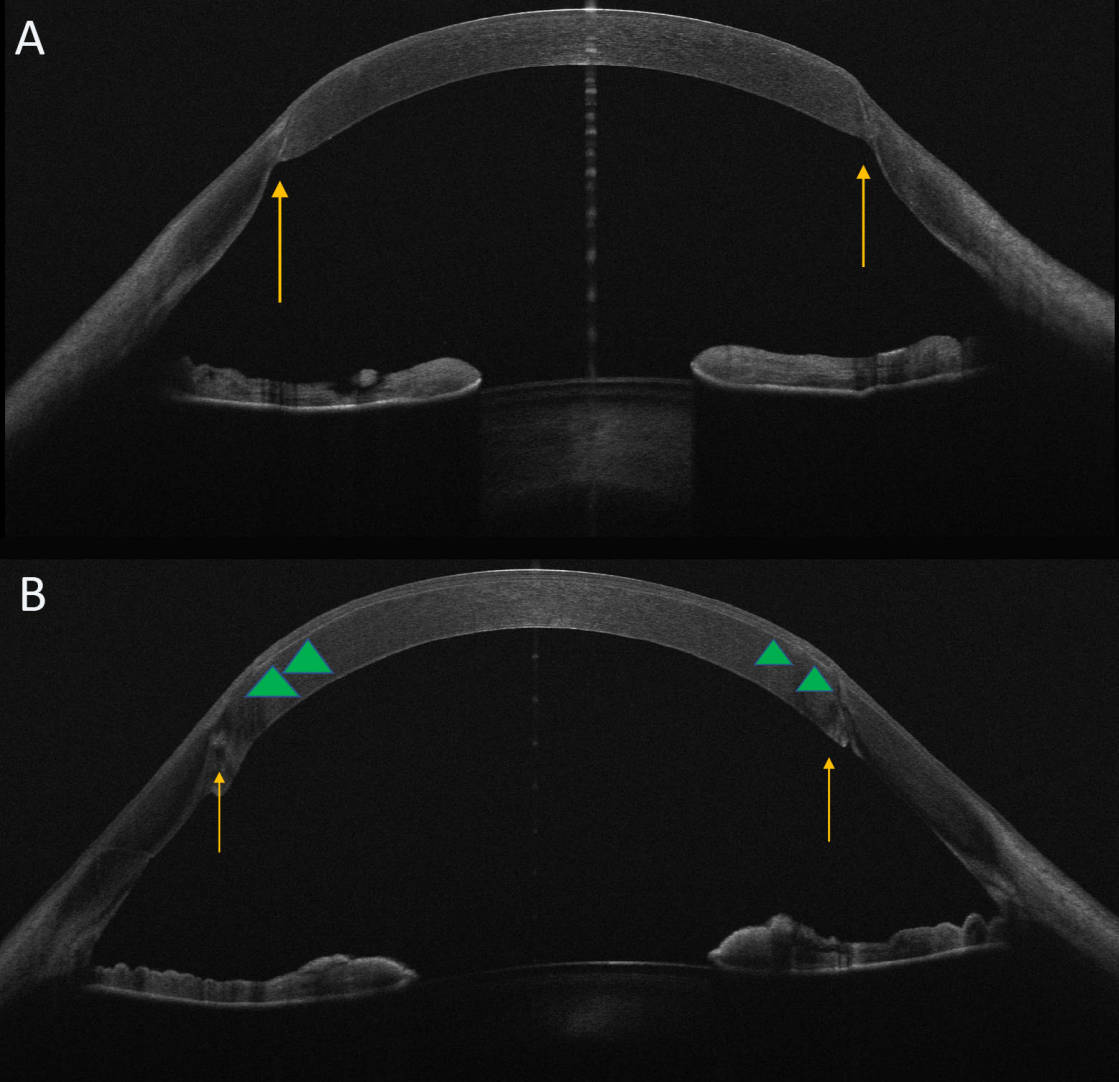
OCT features: pre- and post-refractive treatment. Horizontal OCT scans pre **(A)** and post **(B)** femtoLasik procedure. The images show the graft/host interface (arrows). In particular in Image B OCT scan displays the edge of the flap (arrowheads) as a thin hyper-reflective band.

Microkeratome-LASIK procedure, although it has been widely performed for post-PK refractive errors, is characterized by the risk of complications during the creation of the lamellar flap, such as decentered and free flaps, irregular edges and buttonhole perforations. Furthermore, epithelial trauma and epithelial defects that could occur with mechanical microkeratome can be associated with patient discomfort or photophobia, delayed visual recovery and epithelial ingrowth (9, 17–21, 32). In order to avoid these adverse events, some authors have reported the F-lasik procedure to correct post-PK refractive errors (26, 27) but described few cases and short follow ups. Goreishi et al. (33) described a 12-month follow-up using Ziemer Femto LDV femtosecond laser with a flap of 9 mm in the superior hinge position, and flap thickness was 100 μm for all eyes. Our mean SE reduction and astigmatism from -4.5 ± 2.37 to 1.55 ± 0.77 and from -6.13 ± 2.04 to-3.20 ± 2.15 respectively were statically significant and they were comparable with the results of other studies (26, 33), even though we used a different laser from other authors (**Figure 1**). Changes in SE greater than 0.75-1.00 D and a reduction of at least 1.00-1.50 D in astigmatism are considered clinically significant as they impact visual quality and dependence on corrective lenses. In particular, in our series UCVA improved in all eyes after treatment. More specifically, the mean UCVA improvement was 5 lines. The CDVA showed a statistically significant improvement, but the level of significance is considered weak; this suggests an increase in visual quality, but the improvement is modest compared to other parameters analyzed. The weak significance could be attributed to the small sample size. In detail, CDVA improved in 6 eyes (6/10) whereas it was stable in 4 eyes (4/10). These values are in agreement with previous studies (33).

Refractive outcomes were stable at all follow ups as in previous studies (22, 33), even 3 months after refractive surgery in our series. There were no surgical complications such as corneal wrinkles, epithelial ingrowth, or graft rejection. The limits of our study were the low number of patients included, its retrospective nature, the absence of randomization and, the short follow-up period to show possible regression years later. More specifically, a longer follow-up period (e.g., 3–5 years) would be desirable to fully assess the stability and safety of Femto-LASIK in this patient population. In fact, corneal ectasia (34) is a rare but serious complication that can manifest months or even years after LASIK. Similarly, corneal flap complications, such as displacement or inflammation, may also occur long after the procedure, particularly following ocular trauma. Furthermore, a prospective study could provide us with important information, thanks to, for example, to more standardized procedures. Different treatment approaches have been described in literature, particularly for high refractive errors; in fact, in order to correct residual refractive errors characterized by high astigmatism, some authors evaluated a particular technique using a 2-step treatment, with the combination of arcuate keratotomy and LASIK (29, 34–37): the LASIK flap itself has a significant effect on the astigmatism in post-keratoplasty eyes, therefore, some authors performed excimer laser photoablation as a second step after ensuring refractive stability and reduction of astigmatism. However, other authors preferred one-step F-LASIK procedure due to the risk of developing epithelial ingrowth which can be devastating and might necessitate a new corneal transplantation (26). Furthermore, topography-guided photoablation could be an interesting strategy for non-orthogonal astigmatism, usually observed in grafted patients. Indeed, current devices do not easily collect information from highly irregular surfaces because of light dispersion, diffraction, and aberrations that are too complex for their sensors to read (38, 39). Arcuate keratotomies are effective to improve the mean cylinder, UDVA and CDVA (although not always significantly), but they do not improve the spherical equivalent (7, 40). Corneal wavefront-guided customized LASIK after PKP could be another possible therapeutic approach, although it does not totally correct either refractive errors or high order aberrations (HOAs) due to the high volume of laser ablation required and inadequate corneal stromal thickness. Finally, we have to take into consideration that long-term ectasia in the remaining recipient corneal tissue can occur after PKP in keratoconus patients (41, 42); the delayed refractive instability or recurrent ectasia should be considered when contemplating further surgical refractive procedures in patients who previously have had PKP for keratoconus.

In conclusion, our work confirms previous studies (29, 43) concerning the effectiveness and safety of femto-LASIK treatment for post-PK residual refractive errors particularly in patients with low spherical and cylindrical defects. Reducing astigmatism may permit improved contact lens or spectacle fitting, and therefore achieve best-corrected binocular visual acuity.

## Data Availability

The original contributions presented in the study are included in the article/supplementary material. Further inquiries can be directed to the corresponding author.
